# The immunometabolic axis of sepsis-related myocardial injury: macrophage reprogramming as a central mechanism and therapeutic target

**DOI:** 10.3389/fimmu.2026.1779575

**Published:** 2026-03-11

**Authors:** Yuyang Qiu, Hongying Bi, Wei Xie, Jiao Zhao, Tian Zhang, Jianyu Fu, Xu Liu, Guiyun Li

**Affiliations:** 1 Department of Emergency Intensive Care Unit (EICU), The Second People’s Hospital of Guiyang (Jinyang Hospital)/The Affiliated Jinyang Hospital of Guizhou Medical University, Guiyang, Guizhou, China; 2 Department of Intensive Care Medicine, The Affiliated Hospital of Guizhou Medical University, Guiyang, Guizhou, China

**Keywords:** exosomes, immunometabolism, macrophage polarization, metabolic reprogramming, mitochondria, sepsis-related myocardial injury

## Abstract

Sepsis-related myocardial injury (SRMI) is a major cause of death in critically ill patients, with pathogenesis extending beyond inflammation to encompass dysregulated immunometabolic crosstalk. This review elucidates macrophage metabolic reprogramming as a central mechanism driving SRMI, detailing how a shift to aerobic glycolysis fuels pro-inflammatory responses, while oxidative phosphorylation supports reparative functions. We emphasize that metabolites like succinate, itaconate, and lactate act as potent signaling molecules, orchestrating epigenetic changes and inflammatory pathways. Furthermore, we deconstruct the critical immunometabolic dialogue mediated by extracellular vesicles (EVs) and signaling cascades among macrophages, cardiomyocytes, and endothelial cells. Translating these insights, we evaluate next-generation therapeutic strategies aimed at this immunometabolic axis, including precision small-molecule modulators, nucleic acid-based technologies, and biologics. These approaches represent a promising strategic shift from non-specific immunosuppression toward targeted immunometabolic modulation. This synthesis provides a foundational framework for understanding SRMI and charts a roadmap for developing novel precision medicine interventions to improve patient outcomes.

## 1 Introduction

Sepsis, defined as life-threatening organ dysfunction caused by a dysregulated host response to infection, remains a major challenge in critical care medicine (1, 2). Despite advances in antimicrobial and supportive therapies, sepsis and its complications continue to be a leading cause of global morbidity and mortality (3). Sepsis mortality is strongly associated with the degree of organ dysfunction. Sepsis-related myocardial injury (SRMI), also termed septic cardiomyopathy or sepsis-induced myocardial dysfunction, represents a severe complication and a primary cause of death in sepsis, directly impacting patient prognosis (4, 5). The pathophysiology of SRMI is complex, characterized by inflammatory activation (6, 7), calcium dyshomeostasis (8), oxidative stress (9), and mitochondrial dysfunction (10), collectively contributing to hemodynamic instability and adverse outcomes (5).

### 1.1 Clinical context and evolving phases of sepsis and SRMI

The clinical manifestation of sepsis exists on a continuum of severity. The Sepsis-3 criteria define septic shock as a subset of sepsis characterized by profound circulatory, cellular, and metabolic dysfunction, evidenced by the need for vasopressors to maintain a mean arterial pressure ≥65 mmHg and a serum lactate level >2 mmol/L despite adequate volume resuscitation (1). This distinction is critical, as the profound hypotension and tissue hypoperfusion in septic shock are associated with higher mortality and frequently involve more pronounced myocardial injury. SRMI itself presents heterogeneously across this severity spectrum, ranging from subclinical diastolic dysfunction detectable only by sensitive echocardiographic measures or elevated biomarkers, to overt cardiogenic shock with biventricular dilation and systolic failure (4, 5). The hemodynamic profile may evolve from an early *hyperdynamic* state (characterized by high cardiac output and low systemic vascular resistance) to a late *hypodynamic* state (with low cardiac output) in advanced shock, with microcirculatory dysfunction exacerbating myocardial ischemia and injury (11, 12). Furthermore, the host immune response evolves temporally. An initial hyperinflammatory phase, driven by a surge of pathogen- and damage-associated molecular patterns (PAMPs and DAMPs), is often followed by a protracted immunosuppressive phase marked by immune cell exhaustion and susceptibility to secondary infections (7). The immunometabolic mechanisms underlying SRMI, particularly those involving macrophage reprogramming detailed in this review, are most characteristic of the early hyperinflammatory phase, although maladaptive metabolic processes may persist into later stages, influencing recovery and chronic outcomes.

### 1.2 The heart as an active immunoinflammatory organ

The heart is no longer viewed merely as a passive victim of dysregulated systemic inflammation during sepsis, but as an active participant in the immunoinflammatory response. Stressed and dying cardiomyocytes release DAMPs, including mitochondrial DNA and proteins, which act as potent endogenous ligands to activate innate immune pathways such as the TLRs and cGAS-STING signaling axes in neighboring cells and infiltrating leukocytes (13–15). Furthermore, cardiac-derived extracellular vesicles are increasingly recognized as key vectors for intercellular communication, capable of packaging and disseminating inflammatory signals as well as dysfunctional mitochondrial components, thereby amplifying both local and systemic inflammatory responses (16, 17). This active release of immunogenic signals from the heart establishes a critical, bidirectional dialogue with the immune system, fundamentally shaping the course of sepsis-related myocardial injury.

### 1.3 Central role of macrophages and metabolic reprogramming

Central to this immunoinflammatory response is the macrophage, a functionally versatile sentinel cell of the innate immune system (18, 19). Resident cardiac macrophages and their monocyte-derived counterparts play a dual role: they can exacerbate injury via pro-inflammatory M1 polarization or facilitate repair and inflammation resolution via anti-inflammatory M2 polarization (20). This phenotypic plasticity is increasingly understood to be underpinned by profound metabolic reprogramming (21). The classical pro-inflammatory M1 state is driven by aerobic glycolysis, whereas the anti-inflammatory M2 state primarily relies on oxidative phosphorylation (OXPHOS) and fatty acid oxidation (FAO). This metabolic switch is not merely a consequence of activation but a crucial driver of macrophage function and fate (21).

Recent groundbreaking studies have unveiled the intricate immunometabolic crosstalk within the SRMI. For instance, a specific TREM2-high (TREM2^hi^) resident cardiac macrophage subpopulation has been identified as a key metabolic guardian, essential for clearing damaged mitochondria expelled by cardiomyocytes within extracellular vesicles—a critical process for maintaining myocardial homeostasis during sepsis (22). Conversely, disrupting this delicate balance, for example through TREM2 deficiency, leads to impaired mitochondrial clearance, exacerbated inflammation, and worsened cardiac function (22), highlighting a vital intercellular quality control pathway. Beyond cell-cell interactions, soluble mediators and signaling pathways further illuminate this connection. Research indicates that inhibitors such as BVT.2733 (targeting 11β-hydroxysteroid dehydrogenase type 1, 11β-HSD1) can modulate macrophage polarization via the adenosine 5’-monophosphate (AMP)-activated protein kinase (AMPK)/mammalian target of rapamycin (mTOR) autophagy pathway, thereby ameliorating SRMI (23). Similarly, natural compounds like cichoric acid have demonstrated efficacy in improving SRMI by directly targeting macrophage metabolic reprogramming (24).

### 1.4 Scope and aims of this review

**Figure 1:**
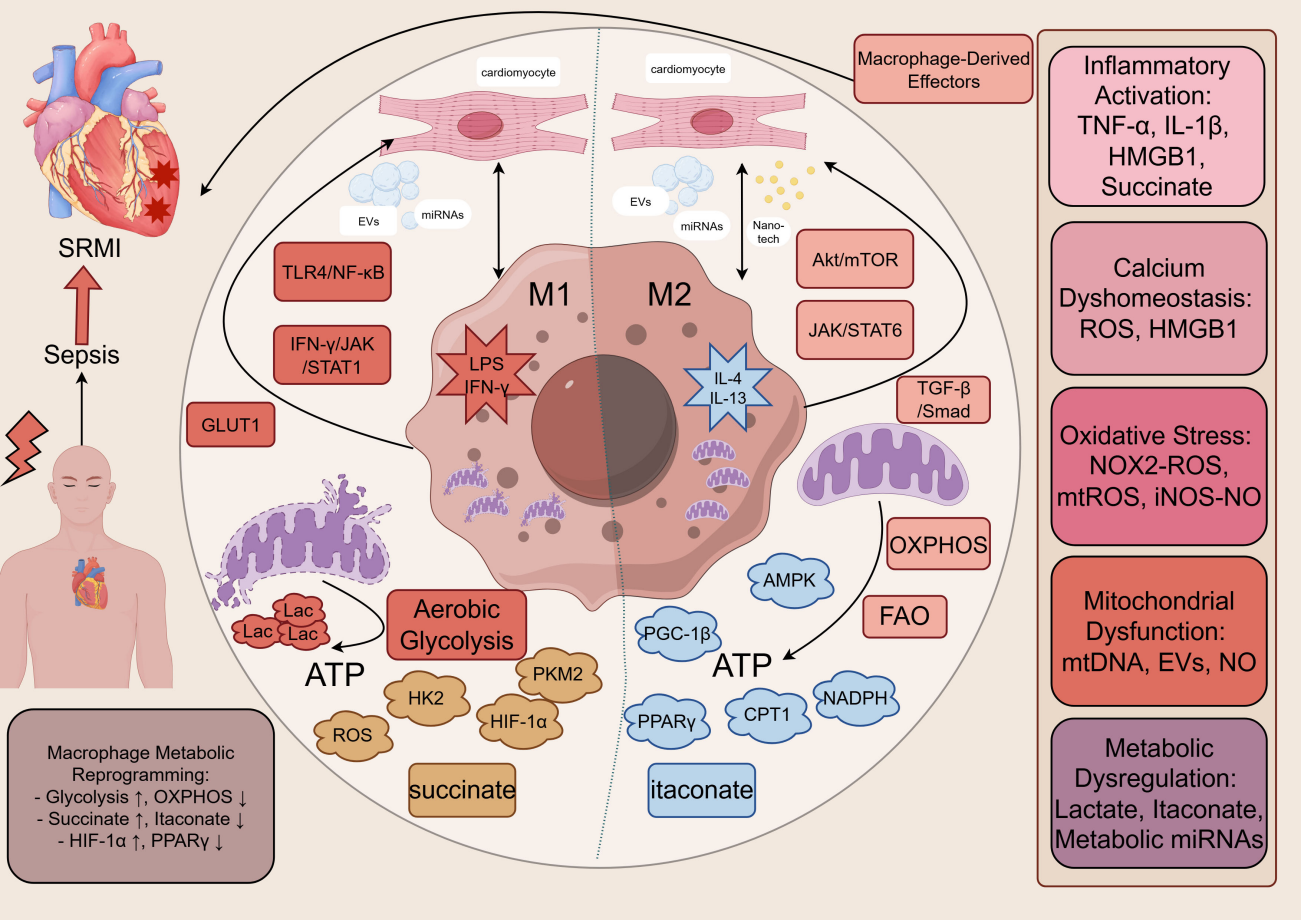
The immunometabolic axis of macrophages in sepsis-related myocardial injury. This schematic illustrates the central, integrative hypothesis of this review. Sepsis (cause) drives profound metabolic reprogramming in cardiac macrophages, characterized by a shift toward aerobic glycolysis in pro-inflammatory states and oxidative metabolism in reparative states. This reprogramming serves as the central mechanism that, through the release of specific cytokines, metabolites, reactive species, and extracellular vesicles, simultaneously engages five core pathophysiological pillars of SRMI: inflammatory activation, calcium dyshomeostasis, oxidative stress, mitochondrial dysfunction, and metabolic dysregulation. For each pillar, the figure delineates the key macrophage-derived effector. The convergence of these parallel yet interconnected pathways culminates in the outcome of myocardial contractile dysfunction and hemodynamic instability. AMPK, AMP-activated Protein Kinase; ATP, Adenosine Triphosphate; CPT1, Carnitine Palmitoyltransferase1; EVs, Extracellular Vesicles; FAO, Fatty Acid Oxidation; GLUT1, Glucose Transporter 1; HIF-1α, Hypoxia-Inducible Factor 1-alpha; HK2, Hexokinase 2; HMGB1, High Mobility Group Box 1; IFN-γ, Interferon-gamma; IL, Interleukin; iNOS, Inducible Nitric Oxide Synthase; JAK, Janus Kinase; Lac, Lactate; LPS, Lipopolysaccharide; miRNA, MicroRNA; mtDNA, Mitochondrial DNA; mtROS, Mitochondrial Reactive Oxygen Species; NADPH, Nicotinamide Adenine Dinucleotide Phosphate Oxidase; NF-κB, Nuclear Factor Kappa-Light-Chain-Enhancer of Activated B Cells; NO, Nitric Oxide; NOX, Nitrogen Oxide; OXPHOS, Oxidative Phosphorylation; PGC-1β, Peroxisome Proliferator-Activated Receptor Gamma Coactivator-1β; PKM2, Pyruvate Kinase Isozyme Type M2; PPARγ, Peroxisome Proliferator-activated Receptor Gamma; ROS, Reactive Oxygen Species; SRMI, Sepsis-Related Myocardial Injury; STAT, Signal Transducer and Activator of Transcription; TGF-β, Transforming Growth Factor-Beta; TNF-α, Tumor Necrosis Factor-alpha; TLR4, Toll-Like Receptor 4.

Despite these advances, a comprehensive review synthesizing how metabolic reprogramming dictates macrophage behavior in SRMI and how this knowledge can be therapeutically harnessed is currently lacking. Existing reviews often focus predominantly on either immunological or metabolic aspects in isolation. This review aims to bridge this gap by integrating the latest evidence on immunometabolic crosstalk in SRMI. We will systematically explore the mechanistic links between metabolic shifts (glycolysis, OXPHOS, FAO) and macrophage polarization, emphasizing the roles of specific metabolites (succinate, itaconate, lactate) and key signaling pathways, like toll-like receptor 4 (TLR4)/nuclear factor kappa-B (NF-κB), AMPK/mTOR, hypoxia-inducible factor 1-alpha (HIF-1α), and peroxisome proliferator-activated receptor γ (PPARγ) (**Figure 1**). We will also systematically discuss the critical mechanisms involving mitochondria and extracellular vesicles (EVs) in promoting macrophage immunometabolism. Furthermore, we will evaluate emerging therapeutic strategies—ranging from pharmacological agents and natural compounds to cellular therapies and nanozymes—that aim to improve SRMI outcomes by targeting macrophage metabolism. By integrating the currently fragmented evidence on macrophage metabolic reprogramming, this review aims to construct a conceptual framework for understanding SRMI, which we anticipate will inform the development of novel therapeutic strategies and stimulate focused investigations in this evolving field.

## 2 The cardiac macrophage landscape in sepsis

### 2.1 Origin and heterogeneity of cardiac macrophages

**Figure 2:**
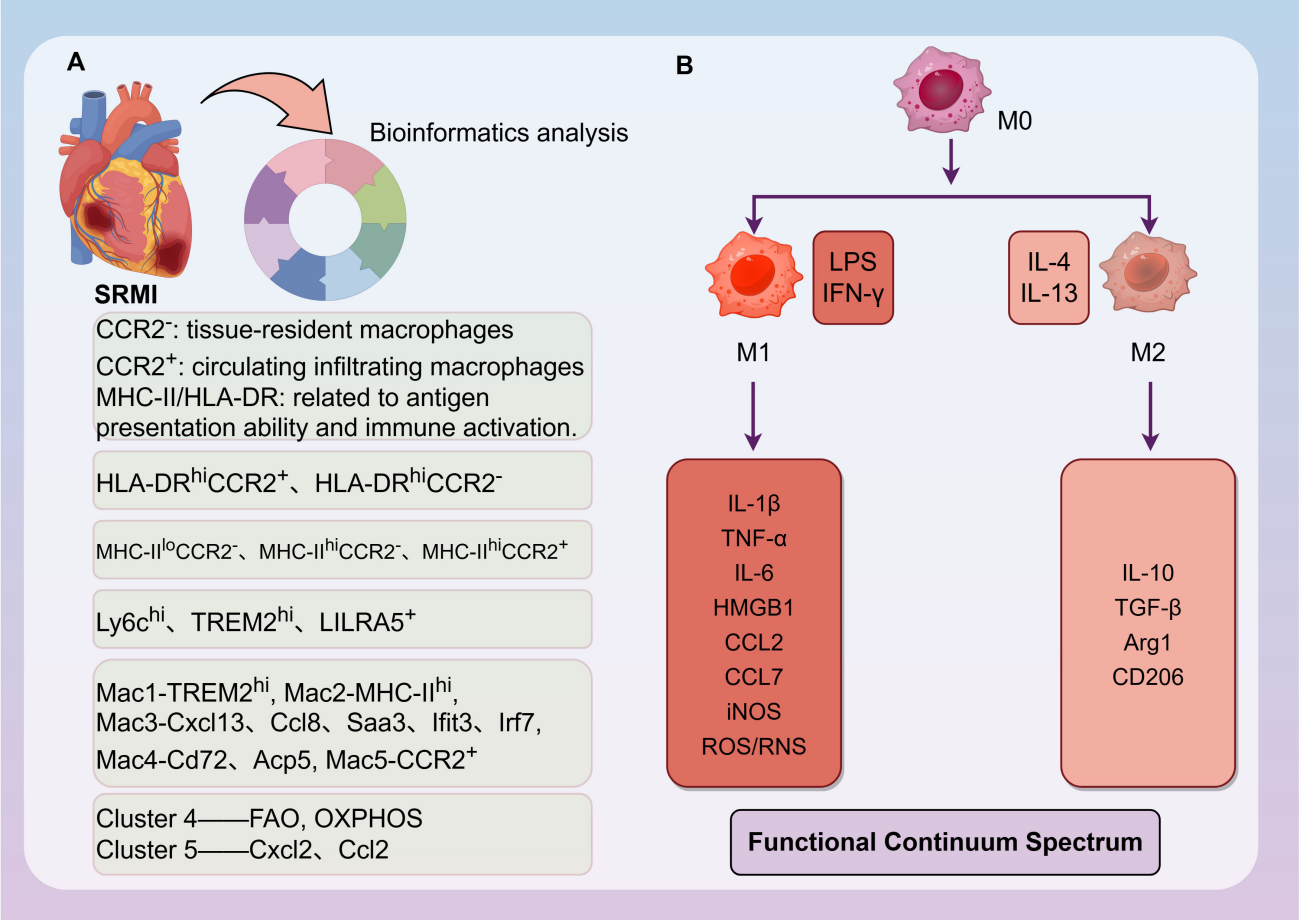
Heterogeneity and polarization dynamics of cardiac macrophages in sepsis. **(A)** Heterogeneity of cardiac macrophage populations in sepsis, as categorized based on markers and functions described in published single-cell transcriptomic studies. The cellular mechanism underlying the cardiac immune response to sepsis (cause) is the expansion of a heterogeneous macrophage landscape. This panel classifies major subsets (e.g., resident TREM2^hi^, infiltrating CCR2^+^, and novel post-sepsis clusters) that emerge, each associated with specific functional phenotypes. **(B)** The polarization mechanism determines functional fate. External signals (cause: e.g., PAMPs like LPS vs. cytokines like IL-4) activate intracellular pathways that mechanistically drive naïve or recruited macrophages toward pro-inflammatory (M1) or anti-inflammatory/reparative (M2) phenotypes. This dynamic switch critically influences the tissue outcome, balancing between inflammatory injury and healing. Arg1, Arginase 1; CCL, C-C Motif Chemokine Ligand; CCR2, C-C Motif Chemokine Receptor 2; FAO, Fatty Acid Oxidation; HLA-DR, Human Leukocyte Antigen DR; HMGB1, High Mobility Group Box 1; IFN-γ, Interferon-gamma; IL, Interleukin; iNOS, Inducible Nitric Oxide Synthase; LPS, Lipopolysaccharide; Mac, Macrophages; MHC-II, Major Histocompatibility Complex Class II; OXPHOS, Oxidative Phosphorylation; RNS, Reactive Nitrogen Species; ROS, Reactive Oxygen Species; SRMI, Sepsis-Related Myocardial Injury; TGF-β, Transforming Growth Factor-Beta; TNF-α, Tumor Necrosis Factor-Alpha; TREM2, Triggering Receptor Expressed on Myeloid Cells 2.

The heart harbors a diverse population of macrophages originating from two primary lineages: embryo-derived resident macrophages and monocyte-derived macrophages recruited from the circulation (25). Under homeostatic conditions, resident macrophages (e.g., CCR2^-^ populations) self-renew and help maintain tissue homeostasis, whereas circulating monocytes (e.g., CCR2^+^ populations) are recruited in response to injury or inflammation (26, 27). Sepsis disrupts this balance: pro-inflammatory cytokines (e.g., TNF-α, IL-1β) and chemokines (e.g., CCL7) drive massive infiltration of monocyte-derived macrophages into the myocardium primarily via the CCL7/CCR2 axis (9). Single-cell transcriptomic studies have further unveiled subpopulations with specialized functions (22, 27–29) (**Figure 2**). The classification of these subsets commonly relies on the markers CCR2 and major histocompatibility complex class II (MHC-II; HLA-DR in humans). CCR2 expression primarily distinguishes the origin and renewal mechanism: CCR2^-^ macrophages are largely embryo-derived resident populations maintained via local proliferation, while CCR2^+^ macrophages are predominantly sustained by monocyte recruitment from the circulation (28). The level of MHC-II/HLA-DR expression is associated with antigen-presenting capacity and immune activation (30). Based on these combined markers, cardiac macrophages are categorized into distinct subsets with different functional propensities. In mice, three main subsets are identified: MHC-II^lo^CCR2^-^, MHC-II^hi^CCR2^-^, and MHC-II^hi^CCR2^+^ (27, 28, 31). In humans, the primary subsets described are HLA-DR^hi^CCR2^-^ and HLA-DR^hi^CCR2^+^ (27, 28, 31). In the context of SRMI, the massive infiltration of CCR2^+^ macrophages drives early inflammatory injury. Conversely, specific resident subsets, such as the MHC-II^hi^CCR2^-^ population in mice, are crucial for maintaining tissue homeostasis and facilitating repair (22).Furthermore, using markers such as MHC-II, Ly6c, CCR2, and CD11c, cardiac macrophages have been classified into four main types (28), which are Ly6c^hi^, MHC-II^hi^CCR2^−^, MHC-II^lo^CCR2^−^, and MHC-II^hi^CCR2^+^ types. A specific TREM2^hi^ resident cardiac macrophage subpopulation has been identified; these TREM2^hi^ macrophages actively clear dysfunctional mitochondria expelled by cardiomyocytes. Conversely, TREM2 deficiency in resident macrophages leads to impaired clearance of damaged mitochondria, resulting in exacerbated cardiac inflammation (22). Specific LILRA5^+^ macrophages contribute to early oxidative stress by producing reactive oxygen species (ROS), further disrupting myocardial structure and function (32). Recent research indicates that following cecal ligation and puncture (CLP) modeling, resident cardiac macrophages proliferate significantly, accompanied by a marked increase in the proportion of the pro-inflammatory MHC-II^hi^CCR2^+^ subtype and a decrease in the MHC-II^lo^CCR2^-^ subtype. Single-cell RNA sequencing of cardiac immune cells after infection resolution (6 weeks) identified two novel macrophage subpopulations (Cluster 4 and 5) almost exclusively present in post-septic hearts (33). Cluster 4 highly expresses genes related to metabolism (e.g., FAO, OXPHOS), while Cluster 5 exhibits significant chemotactic activity, characterized by high expression of neutrophil chemoattractants (e.g., Cxcl2, Ccl2) and inflammatory pathway genes.

### 2.2 Dynamic polarization and functional switch of macrophages

Macrophages directly recognize pathogen-associated molecular patterns (PAMPs) and damage-associated molecular patterns (DAMPs) via surface pattern recognition receptors (PRRs) and opsonin receptors, internalizing them via receptor-mediated endocytosis. This recognition can enhance phagocytic capacity or activate the secretion of pro-inflammatory cytokines (25). Based on their activation mode and function, macrophages are broadly classified into M1 and M2 types (25, 29). Sepsis triggers a profound shift in macrophage polarization, predominantly towards a pro-inflammatory M1 phenotype (34). This is driven by the engagement of Toll-like receptors (TLRs) by PAMPs (e.g., LPS), activating downstream NF-κB signaling and leading to increased expression of cytokines (IL-1β, IL-6, TNF-α) and inducible nitric oxide synthase (iNOS) (29). Concurrently, anti-inflammatory M2 macrophages (e.g., those expressing Arg1, CD206, TGF-β) are suppressed, impairing tissue repair processes (29, 35). However, the M1/M2 dichotomy represents two extremes of a continuum; in reality, macrophage polarization exists along a spectrum rather than a strict binary classification. Although the M1/M2 framework provides a simplified model, its limitations in explaining and treating complex diseases have prompted the adoption of more refined subpopulation analyses, providing a critical foundation for personalized precision medicine (25).

### 2.3 Mechanisms of macrophage-mediated myocardial injury

Activated macrophages exacerbate SRMI through multiple, highly synergistic molecular mechanisms that extend far beyond simple cytokine release. These mechanisms involve complex intercellular communication, metabolic disruption, and the induction of programmed cell death (5, 36).

#### 2.3.1 Inflammatory cytokines and myocardial suppression

During sepsis, macrophages sensing PAMPs via PRRs (e.g., TLR4) activate key signaling pathways such as NF-κB and mitogen-activated protein kinase (MAPK), driving the synthesis and release of large quantities of pro-inflammatory cytokines (TNF-α, IL-1β, IL-6) (36–38). These cytokines contribute to myocardial dysfunction by activating downstream signaling pathways that collectively impair cardiomyocyte contractility (38, 39). The major mechanisms include: (i) disruption of calcium handling, through effects on sarcoplasmic reticulum Ca^2+^ release and reuptake, leading to abnormal Ca^2+^ transients; (ii) desensitization of β-adrenergic signaling, reducing the cardiomyocyte’s inotropic response to sympathetic stimulation; (iii) induction of iNOS, resulting in sustained high-level NO production that can suppress mitochondrial respiration and modify contractile proteins; and (iv) promotion of mitochondrial dysfunction, compromising ATP generation essential for contraction and relaxation (37–39). High mobility group box 1 (HMGB1), a critical late inflammatory mediator, is actively released by activated macrophages or passively spilled from necrotic cells. It mediates sepsis progression, binds to TLR4 to promote further cytokine production, exacerbates tissue inflammatory injury (40, 41), and may play a mediating role in the pathophysiology of sepsis-induced cardiomyopathy (5).

#### 2.3.2 Calcium dyshomeostasis and disrupted excitation-contraction coupling

Precise temporal and spatial control of intracellular Ca^2+^ cycling is fundamental to cardiomyocyte excitation-contraction coupling. In SRMI, this delicate balance is profoundly disrupted, contributing directly to both systolic and diastolic dysfunction. Macrophage-derived inflammatory mediators are central instigators of this dyshomeostasis, targeting several key molecular nodes within the cardiomyocyte.

The sarcoplasmic reticulum (SR) is a critical reservoir for Ca^2+^. Its release through the ryanodine receptor 2 (RyR2) channel and reuptake via the sarcoplasmic/endoplasmic reticulum calcium ATPase 2a (SERCA2a) are tightly regulated processes. Sepsis-associated inflammation leads to pathological “leakiness” of RyR2 and impaired SERCA2a activity. Pro-inflammatory cytokines such as TNF-α and IL-1β can promote the hyperphosphorylation of RyR2, for instance via protein kinase A (PKA) and calcium/calmodulin-dependent protein kinase II (CaMKII), increasing its open probability during diastole and depleting SR Ca^2+^ stores (42). Concurrently, cytokine signaling and oxidative stress downregulate SERCA2a expression and activity, slowing Ca^2+^ reuptake and impairing relaxation (43). The resulting reduction in SR Ca^2+^ content diminishes the amplitude of the subsequent systolic Ca^2+^ transient, directly compromising contractile force.

Reactive oxygen and nitrogen species (ROS/RNS) from activated macrophages further exacerbate calcium handling defects. Oxidative modification of RyR2 and SERCA2a proteins can alter their function directly. Moreover, sustained intracellular ROS activates CaMKII, which not only phosphorylates RyR2 but also contributes to maladaptive transcriptional changes (44). The high levels of nitric oxide produced via macrophage-induced iNOS can lead to S-nitrosylation of RyR2, potentially contributing to diastolic leak, while also inhibiting mitochondrial respiration, reducing the ATP available to fuel SERCA2a and other ATP-dependent processes (45).

The DAMP HMGB1, released from activated macrophages, is a potent disruptor of cardiomyocyte Ca^2+^ homeostasis. By binding to TLR4 on cardiomyocytes, HMGB1 activates downstream ROS production within the myocyte. This intracellular ROS signaling has been shown to enhance RyR2-mediated SR Ca^2+^ leak, creating a paracrine loop of dysfunction originating from the immune cell (5, 40).

Mitochondrial calcium overload is another consequential event. As major sinks for cytosolic Ca^2+^ during systole, mitochondria take up Ca^2+^ via the mitochondrial calcium uniporter (MCU) to stimulate ATP production. However, under conditions of excessive cytosolic Ca^2+^ and oxidative stress—both promoted by the inflammatory milieu—this uptake can become pathological. Mitochondrial Ca^2+^ overload promotes the opening of the mitochondrial permeability transition pore (mPTP), leading to loss of membrane potential, swelling, and the release of pro-apoptotic factors, thereby linking calcium dysregulation directly to mitochondrial dysfunction and cell death (46).

#### 2.3.3 Mitochondrial dysfunction

Mitochondrial dysfunction within macrophages themselves represents another crucial pathway amplifying myocardial injury. LPS stimulation can induce massive production of mitochondrial reactive oxygen species (mtROS). mtROS acts not only as a destructive oxidant but also as a significant signaling molecule, regulating IL-1β release and pyroptosis, thereby further amplifying the inflammatory cascade and impacting neighboring cardiomyocytes (47). Furthermore, recent studies indicate that NF-κB-mediated upregulation of phospholipid metabolism in sepsis elevates HIF-1α, which subsequently induces excessive iNOS-dependent NO production, inhibiting mitochondrial respiratory chain complexes I-III activity. This triggers mitochondrial dysfunction and cytopathic hypoxia, ultimately contributing to cardiac contractile failure (45).

#### 2.3.4 Induction of ferroptosis

Emerging evidence suggests a link between macrophage metabolic reprogramming and the induction of ferroptosis, presenting a potential novel axis in the pathogenesis of SRMI (36, 48). Activated macrophages appear to create a pro-ferroptotic microenvironment by undergoing a metabolic shift characterized by upregulated acyl-CoA synthetase long-chain family member 4 (ACSL4), which increases the pool of peroxidizable phospholipids, and downregulated glutathione peroxidase 4 (GPX4), the key enzyme responsible for lipid peroxide repair (36, 49, 50). This ACSL4/GPX4 imbalance promotes lethal lipid peroxidation. Supporting this pathophysiological concept, interventions that mitigate ferroptosis have been shown to confer cardiac protection in experimental sepsis (51). Therefore, macrophage-mediated facilitation of ferroptosis is increasingly implicated as a contributing mechanism to myocardial damage in sepsis, warranting further investigation to fully elucidate its precise role (36). Beyond ferroptosis, other regulated cell death pathways contribute to inflammatory tissue injury. Necroptosis, a programmed form of necrosis mediated by RIPK1, RIPK3, and MLKL, is activated by TNF-α and other inflammatory signals abundant in sepsis. While direct evidence in SRMI is evolving, necroptosis is a well-established driver of inflammation and adverse remodeling in other forms of myocardial injury (52, 53). Necroptosis is also closely related to the activation state of macrophages during cardiac-related diseases. Its potential role in SRMI, potentially contributing to cardiomyocyte loss and DAMP release, warrants further investigation.

#### 2.3.5 Amplification of oxidative and nitrosative stress

In SRMI, oxidative and nitrosative stress arise from a concerted overproduction of ROS/RNS across multiple cardiac cell types. Macrophages, particularly subsets like LILRA5^+^ cells, are early instigators, generating superoxide via cytosolic NADPH oxidase (NOX2) and mitochondrial ROS (mtROS) via electron transport chain leakage (32, 47). Cardiomyocytes themselves become significant ROS sources as sepsis-induced mitochondrial injury creates a self-amplifying cycle of mtROS production. Concurrently, endothelial dysfunction contributes to oxidative stress through uncoupled endothelial nitric oxide synthase (eNOS), which generates superoxide instead of protective nitric oxide (NO) (54). The major RNS, NO, is produced at high, sustained levels by inducible NOS (iNOS) in immune and cardiac cells. This pathological NO, and its potent derivative peroxynitrite (ONOO^−^), inhibit mitochondrial respiratory complexes, contributing to bioenergetic failure or “cytopathic hypoxia” (45).

The cardiac-specific pathophysiology stems from the precise targeting of these reactive species to critical cardiomyocyte components. ROS and RNS directly impair excitation-contraction coupling by oxidizing key cysteine residues on the sarcoplasmic reticulum calcium release channel (RyR2), promoting diastolic leak, and by inhibiting the calcium reuptake pump (SERCA2a) (55, 56). They also sensitize the mPTP to calcium-dependent opening, triggering loss of mitochondrial integrity and bioenergetic collapse (57). Furthermore, oxidative modifications of myofilament proteins (e.g., troponin I) reduce calcium sensitivity and contractile force (58). At the microcirculatory level, endothelial-derived ROS and a deficit in bioactive NO exacerbate ischemia by impairing vasodilation and increasing permeability, creating a synergistic loop between vascular dysfunction and myocardial injury (12).

## 3 Fundamentals of macrophage metabolic reprogramming

**Figure 3:**
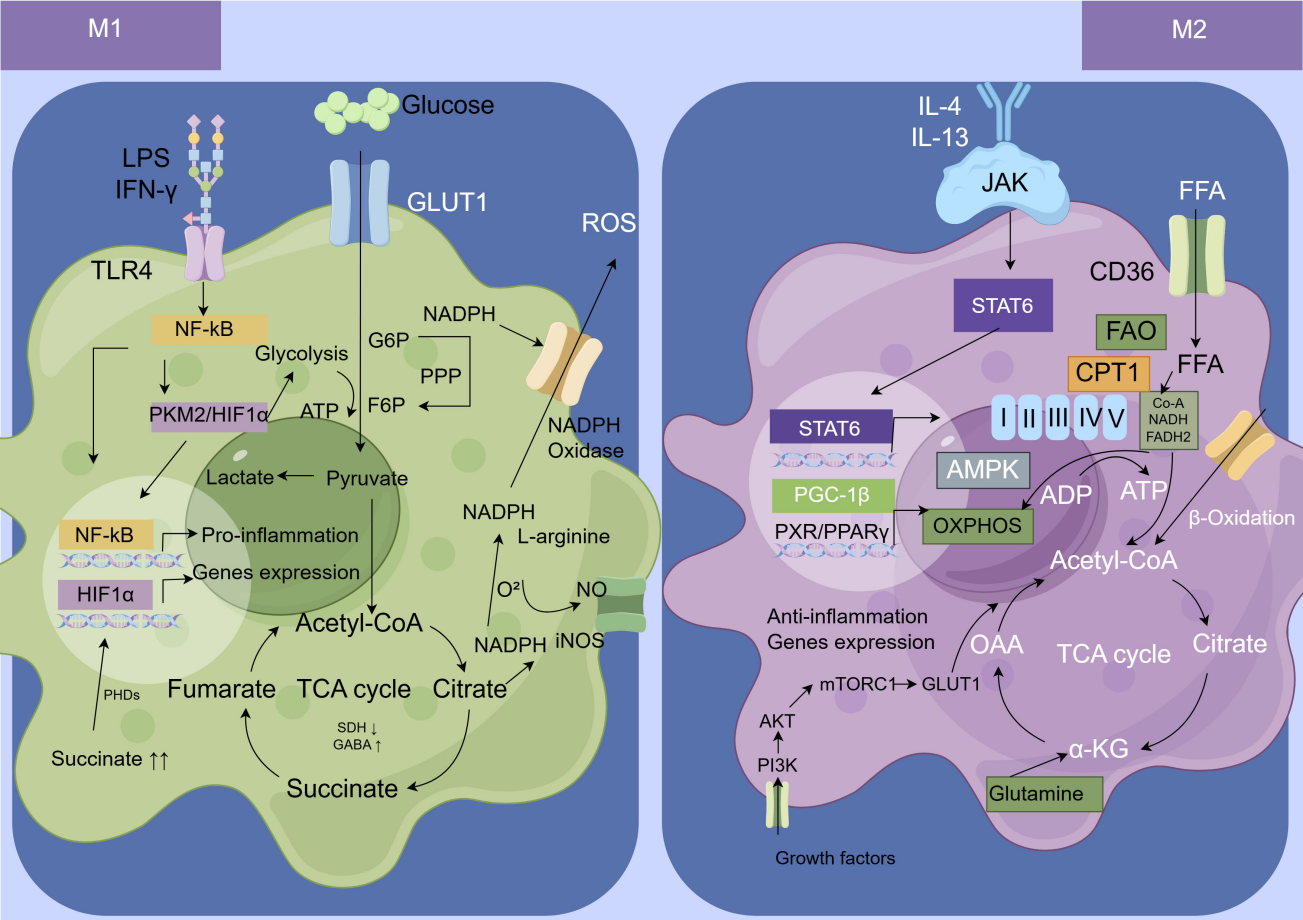
Metabolic reprogramming underpinning macrophage M1 and M2 polarization. This figure contrasts the specific metabolic mechanisms that enact macrophage polarization. Distinct stimuli (cause) trigger a mechanistic rewiring of core pathways: inflammatory signals promote aerobic glycolysis and a broken TCA cycle in M1 cells, while anti-inflammatory signals enhance oxidative phosphorylation and fatty acid oxidation in M2 cells. These divergent metabolic programs directly power and define their respective pro-inflammatory or anti-inflammatory/reparative functional phenotypes. AMPK, AMP-activated Protein Kinase; ADP, Adenosine Diphosphate; ATP, Adenosine Triphosphate; α-KG, α-Ketoglutaric Acid; CPT1, Carnitine Palmitoyltransferase1; FADH2, Flavine Adenine Dinucleotide, Reduced; FAO, Fatty Acid Oxidation; FFA, Free Fat Acid; GABA, Gama-Aminobutyric Acid; GLUT1, Glucose Transporter 1; HIF-1α, Hypoxia-Inducible Factor 1-alpha; IFN-γ, Interferon-gamma; IL, Interleukin; iNOS, Inducible Nitric Oxide Synthase; JAK, Janus Kinase; LPS, Lipopolysaccharide; NADH, Nicotinamide Adenine Dinucleotide; NADPH, Nicotinamide Adenine Dinucleotide Phosphate Oxidase; NF-κB, Nuclear Factor Kappa-Light-Chain-Enhancer of Activated B Cells; NO, Nitric Oxide; OAA, Oxaloacetic Acid; OXPHOS, Oxidative Phosphorylation; PGC-1β, Peroxisome Proliferator-Activated Receptor Gamma Coactivator-1β; PHDs, proline hydroxylases; PI3K, Phosphatidyqinositol‐3 Kinase; PKM2, Pyruvate Kinase Isozyme Type M2; PPARγ, Peroxisome Proliferator-Activated Receptor Gamma; PPP, Pentose Phosphate Pathway; ROS, Reactive Oxygen Species; SDH, Succinate Dehydrogenase; STAT, Signal Transducer and Activator of Transcription; TCA, Tricarboxylic Acid; TLR4, Toll-Like Receptor 4.

The immunometabolism of macrophages primarily involves key metabolic pathways, including aerobic glycolysis, the tricarboxylic acid (TCA) cycle, OXPHOS, fatty acid metabolism, and amino acid metabolism. Metabolic reprogramming is a fundamental process underpinning the functional plasticity of macrophages, enabling them to respond to environmental signals. This reprogramming entails dynamic shifts in metabolic pathways that not only fulfill energy and biosynthetic demands but also directly influence inflammatory gene expression and cell fate through metabolic intermediates that function as signaling molecules. Underpinning this functional plasticity are both these metabolic shifts and a set of key signaling pathways. The TLR4/NF-κB and IFN-γ/JAK/STAT1 pathways are central to M1 polarization, while the PI3K/Akt/mTOR, TGF-β/Smad, and IL-4/JAK/STAT6 pathways are pivotal for M2 polarization (25) (**Figure 3**).

### 3.1 Metabolic characteristics of the pro-inflammatory M1 phenotype

Activation of macrophages by LPS and IFN-γ triggers a reprogramming of glucose metabolism, shifting the cellular energy production method from OXPHOS towards aerobic glycolysis. This phenomenon, analogous to the Warburg effect observed in cancer cells, allows for the rapid, high-yield generation of energy and substrates within a short timeframe to meet the biosynthetic needs of activated macrophages (59, 60). Enhanced glucose uptake and a shift towards glycolysis are hallmark features of M1 macrophages, whereas enhanced FAO and OXPHOS are characteristic of M2 macrophages (61, 62). The rapid increase in glycolytic flux is facilitated by the upregulation of key enzymes and transporters, including glucose transporter 1 (GLUT1), hexokinase 2 (HK2), and the M2 isoform of pyruvate kinase (PKM2) (21, 63, 64). PKM2, which regulates HIF-1α activity and IL-1β induction, is a key determinant of the Warburg effect in LPS-activated macrophages (21, 63). HIF-1α plays a crucial role as a key hub connecting metabolism, immunity and inflammatory responses in septic macrophages. The normal functioning of HIF-1α directly affects the pathological process and prognosis of sepsis. Reprogramming of the TCA cycle in M1 macrophages is characterized by inhibited succinate dehydrogenase (SDH) activity and an upregulated gamma-aminobutyric acid (GABA) shunt, leading to substantial succinate accumulation. Succinate stabilizes HIF-1α by inhibiting prolyl hydroxylases (PHDs), thereby further amplifying glycolysis and IL-1β production (65, 66). There is also an increased efflux of citrate from mitochondria to the cytosol, mediated by the mitochondrial citrate carrier which exchanges citrate for malate. Cytosolic citrate is then utilized for fatty acid biosynthesis (62, 65). Furthermore, citrate accumulation can contribute to the production of prostaglandins, NO, and ROS, exerting pro-inflammatory effects (62). Beyond succinate, two other TCA cycle-related metabolites, itaconate and fumarate, have been demonstrated to play critical and complex roles in macrophage-mediated immune responses and inflammatory diseases (67). GLUT1, the rate-limiting glucose transporter in M1 macrophages, exhibits increased expression that contributes to ROS production and pro-inflammatory mediator expression (68). Enhanced activity of the pentose phosphate pathway (PPP) generates NADPH, which is utilized for the production of ROS and NO via NADPH oxidase and iNOS, respectively (69).

### 3.2 Metabolic characteristics of the anti-inflammatory M2 phenotype

Anti-inflammatory M2 macrophages, activated by IL-4 and IL-13, exhibit a metabolic profile centered on OXPHOS and FAO. Promoting FAO enhances the anti-inflammatory function of M2 macrophages, while its inhibition diminishes their anti-inflammatory effects (62, 69). The glucose metabolism of M2 macrophages primarily relies on OXPHOS and mitochondrial respiration for energy production, a process regulated by the transcription factor STAT6 (70). Additionally, AMPK acts as a key regulator of OXPHOS and influences M2 polarization by promoting catabolism, inhibiting anabolism, and modulating mitochondrial biogenesis (71). mTOR, which integrates nutrient sensing with glucose metabolism, lipid metabolism, and biosynthesis, is one of the key regulators of metabolic activation in macrophages (70). The transcription factor PPARγ plays a vital role in regulating cell proliferation, differentiation, and immune responses, and is involved in lipid, glucose, and amino acid metabolism, promoting adipocyte differentiation and enhancing glucose uptake (72). PPARγ promotes the expression of anti-inflammatory mediators (e.g., IL-4, CD163, adiponectin) in macrophages while simultaneously suppressing genes encoding pro-inflammatory molecules (e.g., TNF-α, IL-1β, IL-6) (72, 73). In macrophages, the upregulated FAO pathway breaks down fatty acids into acetyl-CoA, provides electron donors (NADH, FADH2) for the mitochondrial electron transport chain, and feeds into the TCA cycle and OXPHOS (74, 75). The rate-limiting step of FAO is the conjugation of fatty acids with carnitine by carnitine palmitoyltransferase 1 (CPT1). In M2 macrophages, FAO is increased under the regulation of PGC1β or AMPK; inhibition of CPT1 suppresses the IL-4-induced increase in FAO and limits M2 activation (74). Increased glutamine metabolism is another key feature, wherein glutamine is converted to α-ketoglutarate (α-KG), contributing to the replenishment of TCA cycle intermediates and the generation of UDP-GlcNAc. The latter promotes the glycosylation of lectin/mannose receptors, which are typical M2 polarization markers (69, 75). Furthermore, M2 macrophages express glutamine synthetase (GS), which plays a positive role in M2 polarization (e.g., IL-10 secretion) (69). Some studies suggest that glycolysis may also be crucial for the differentiation of M2 macrophages (76). A potential mechanistic link involves pyruvate generated from glycolysis entering the TCA cycle to promote acetyl-CoA synthesis and histone acetylation or mitochondrial OXPHOS. Additionally, pharmacologic activation of PKM2 using TEPP-46 was shown to increase the production of the anti-inflammatory cytokine IL-10 in LPS-activated macrophages via metabolic reprogramming, leading to ATP generation and release derived from glycolysis (77). However, another study investigating this effect indicated that stimulating glycolysis is not necessary for M2 macrophage differentiation as long as OXPHOS remains active (61).

### 3.3 Integrating macrophage metabolic reprogramming with the core pathophysiological pillars of SRMI

The pathophysiology of SRMI converges on several interrelated pillars: inflammatory activation, calcium dyshomeostasis, oxidative stress, mitochondrial dysfunction, and metabolic derangements, collectively leading to hemodynamic instability (5). Macrophage metabolic reprogramming is not a parallel event but a central upstream mechanism that drives these core pathological processes. This section provides a systematic integration of these concepts (**Figure 1**).

#### 3.3.1 Inflammatory activation

The engagement of macrophage pattern recognition receptors by PAMPs triggers a dual inflammatory-metabolic response. Signaling via NF-κB and MAPK pathways drives pro-inflammatory cytokine gene expression (37), while a concurrent shift to aerobic glycolysis stabilizes HIF-1α, which further amplifies the transcription of cytokines like IL-1β (63, 66). Critically, this metabolic reprogramming itself generates inflammatory signals. The accumulation of succinate inhibits prolyl hydroxylases, reinforcing HIF-1α stability, and can drive mitochondrial reverse electron transport to generate ROS that activate the NLRP3 inflammasome (47, 66). Thus, macrophage metabolism directly fuels the inflammatory cascade, creating a feed-forward loop that sustains cytokine release and contributes to myocardial depression (38, 39).

#### 3.3.2 Calcium dyshomeostasis

This pillar is significantly exacerbated by macrophage-derived inflammatory mediators and reactive species. Beyond direct cytokine effects on cardiomyocyte calcium channels and pumps (37, 39), macrophage metabolic activity plays a key role. For instance, the release of HMGB1 from activated macrophages binds to TLR4 on cardiomyocytes, activating intracellular ROS signaling that increases diastolic calcium leak from the sarcoplasmic reticulum (5, 40). Furthermore, sustained oxidative stress from macrophage activity can promote post-translational modifications of critical calcium-handling proteins like RyR2 and SERCA2a, disrupting calcium transient kinetics and directly impairing excitation-contraction coupling (8).

#### 3.3.3 Oxidative stress

Macrophages are pivotal instigators of cardiac oxidative stress in sepsis. Specific subsets, such as LILRA5+ macrophages, are early and potent sources of ROS via NOX2 and mitochondrial leakage (32). The metabolic shift to glycolysis and an active pentose phosphate pathway further augments the NADPH pool available for ROS generation. This macrophage-derived oxidative burst directly damages cardiomyocyte structural and functional proteins. Importantly, it also targets the coronary microvascular endothelium, increasing permeability and activating inflammatory adhesion molecules, which compromises tissue perfusion and creates a secondary ischemic insult that exacerbates global cardiac dysfunction (12).

#### 3.3.4 Mitochondrial dysfunction

Macrophages contribute to mitochondrial failure in cardiomyocytes through both indirect and direct mechanisms. The induction of iNOS leads to excessive NO production, which inhibits mitochondrial respiratory chain complexes (45). Moreover, a critical protective dialogue is disrupted when resident cardiac macrophage function is impaired. TREM2^hi^ macrophages normally phagocytose and clear mitochondria ejected by stressed cardiomyocytes via extracellular vesicles (22). Failure of this clearance mechanism, due to either macrophage dysfunction or loss, leads to the accumulation of damaged mitochondria within the myocardium. These dysfunctional organelles release mtDNA and produce excessive mtROS, activating cardiomyocyte inflammasomes and creating a vicious cycle of bioenergetic crisis and inflammation.

#### 3.3.5 Metabolic alterations

The SRMI faces a profound metabolic crisis, and macrophages are active participants in shaping this environment. Their preferential use of glycolysis elevates local lactate levels, which can serve as a signaling molecule influencing histone lactylation and gene expression in neighboring cells (78). Conversely, the production of itaconate in certain macrophage subsets can exert paracrine protective effects on cardiomyocytes by activating the Nrf2 antioxidant pathway (79). The exchange of metabolites and regulatory miRNAs via exosomes further underscores how macrophage immunometabolism directly modulates the cardiac metabolic landscape, affecting substrate utilization and energy resilience.

In summary, macrophage metabolic reprogramming acts as a central conductor, orchestrating multiple pathological processes in SRMI. By generating specific metabolic intermediates, redox signals, and paracrine factors, it simultaneously engages the inflammatory, calcium handling, oxidative, mitochondrial, and metabolic pillars of disease. This integrated perspective underscores why targeting macrophage metabolism presents a promising therapeutic strategy with multi-faceted potential.

## 4 Immunometabolic crosstalk in sepsis-related myocardial injury

SRMI is not an isolated event involving a single cell type or pathway, but rather the result of complex crosstalk, mediated by metabolic signals, among cardiomyocytes, immune cells (particularly macrophages), and vascular endothelial cells (5, 80). This “immunometabolic dialogue” constitutes a core network in the pathological progression of SRMI (67). This section will systematically elaborate on how metabolite signaling, mitochondrial dysfunction, and intercellular communication collectively drive myocardial injury (**Table 1**).

**Table 1:** Key immunometabolic crosstalk mechanisms in sepsis-related myocardial injury.

Mechanism	Key participants/signals	Core function/effect	Impact on cardiac function	Key supporting references
Metabolite Signaling	Succinate, Itaconate, Lactate, Histone Lactylation (H3K18la)	Regulates HIF-1α/Nrf2 activity; mediates epigenetic modifications.	Drives or suppresses inflammatory responses; reprograms gene expression.	(66, 67, 78, 79, 81, 84)
Mitochondrial Quality Control	TREM2^hi^ Macrophages, Extracellular Vesicles, Mitophagy	Clears damaged mitochondria; maintains metabolic homeostasis.	Protects cardiomyocytes from oxidative stress and cell death.	(22, 47, 75)
Intercellular Communication	Exosomes (miRNAs), Ca^2+^/CaMKIIα pathway	Facilitates remote cell communication; regulates inflammasome activity.	Coordinates the integrated immunometabolic response of the heart.	(8, 95, 97)
Microcirculation & Endothelium	Cardiac Microvascular Endothelial Cells, Barrier regulation (e.g., HSP90)	Maintains microvascular integrity; dysregulation amplifies injury.	Impairs tissue perfusion, exacerbating hypoxia and metabolic dysregulation.	(11, 12, 104, 105)
Regulated Cell Death & Emerging Regulators	Ferroptosis (ACSL4/GPX4), Necroptosis, Epitranscriptomic regulation (e.g., m6A)	Contributes to cardiomyocyte loss and inflammation; offers novel regulatory layers.	Reduces contractile mass; modulates injury and repair responses.	(36, 49, 51–53, 108, 109)

This table provides a concise summary of the central immunometabolic dialogue driving myocardial injury in sepsis, outlining core mechanisms, key participants, their pathophysiological functions, and ultimate impact on cardiac function. It serves as a synthesized reference for the principal pathways discussed in this review. The numbers listed under the “Key Supporting References” column represent the order of the cited references in the main text.

ACSL4, Acyl-CoA Synthetase Long Chain Family Member 4; CaMKIIα, Ca^2+^/calmodulin-dependent protein kinase II alpha; GPX4, Glutathione Peroxidase 4; HIF-1α, Hypoxia-Inducible Factor 1-alpha; H3K18la, Histone Lactylation at Lysine 18; HSP90, Heat shock protein 90; m6A, N6-methyladenosine; Nrf2, Nuclear Factor Erythroid 2–Related Factor 2; TREM2, Triggering Receptor Expressed on Myeloid Cells 2.

### 4.1 Metabolites as signaling molecules: bridging metabolic status and immune response

In sepsis, rapidly shifting metabolic states directly regulate the gene expression and function of immune cells via specific metabolites (67). For instance, succinate accumulation in LPS-activated M1 macrophages not only inhibits PHDs, thereby stabilizing HIF-1α and enhancing the transcription of pro-inflammatory factors like IL-1β (81), but also drives reverse electron transport (RET) via SDH oxidation, leading to a burst of ROS (66). In contrast, itaconate, a metabolite associated with M2 macrophages, inhibits SDH, reduces the secretion of pro-inflammatory cytokines (e.g., IL-1β, IL-18, IL-12, IL-6) (79), and exerts anti-inflammatory effects by blocking SDH-mediated RET and ROS generation, thereby inhibiting HIF-1α accumulation (66). Furthermore, itaconate can activate the Nrf2 antioxidant pathway by alkylating KEAP1 (82). In a murine model of SRMI, cardiac-specific heterozygous deletion of HIF-1α, acting through an NF-κB-mediated pathway involving COX2 and secretory phospholipase A2 (sPLA2), improved mitochondrial dysfunction and myocardial contractile dysfunction (83). Lactate, the end product of glycolysis, is significantly elevated in the SRMI. Research indicates that endogenous, macrophage-derived lactate can be metabolized to acyl-CoA (lactoyl-CoA), leading to direct histone lactylation, which promotes the functional repolarization of LPS/IFN-γ-induced M1 macrophages towards an M2-like phenotype (78). Another study found that IL-4-induced polarization of M0 macrophages to M2 macrophages was accompanied by an interchangeable glucose- or lactate-dependent TCA cycle metabolism, which directly drives histone acetylation, M2 gene transcription, and functional immunosuppression, representing an important epigenetic modification (84). Studies also show that lactate is a primary source for acetyl-CoA generation in trained monocytes, directly driving histone lactylation. Lactate dehydrogenase A (LDHA), crucial in lactate metabolism by regulating the lactate/pyruvate interconversion, is essential both for lactate-driven TCA cycling and for histone lactylation (85). Collectively, these studies demonstrate that macrophage metabolites act not merely as energy substrates but also as potent signaling molecules regulating gene expression, playing a pivotal role in bridging metabolic status with the immune response.

### 4.2 Mitochondria: the central hub of immunometabolic crosstalk

Mitochondria serve as both the cellular powerhouses and critical nodes of immunometabolic crosstalk in the SRMI (86). Mitochondrial biogenesis, fusion, and fission play roles in immune cell activation and are central to the immune system (75). Sepsis can induce mitochondrial dysfunction in cardiomyocytes, characterized by impaired electron transport chain activity and excessive ROS production (87). Mitochondrial DNA (mtDNA), encoding key components of the respiratory chain, is vital for mitochondrial function. Misincorporation of ribonucleotides into mtDNA can disrupt its integrity and function, triggering a cascade of events culminating in inflammation (88). mtDNA acts as a DAMP, and the mitochondrial outer membrane serves as a platform for signaling molecules like MAVS in the RIG-I pathway and as the location for the NOD-like receptor protein 3 (NLRP3) inflammasome. A key aspect of mitochondrial signaling is the generation of mtROS, which function as crucial second messengers in both adaptive and innate immune regulation, participating in the M1/M2 polarization of macrophages and determining the type and intensity of the immune response (89). mtROS produced by LPS-stimulated macrophages act as antimicrobial agents and redox signals; mtROS generated via RET regulate IL-1β release during NLRP3 inflammasome activation (47).

#### 4.2.1 TREM2^hi^ macrophages and the intercellular mitochondrial clearance pathway

The discovery of cardiac-resident TREM2^hi^ macrophages has elucidated a sophisticated intercellular mechanism for mitochondrial quality control in the SRMI, comprising mitochondrial export, recognition, and phagocytic clearance. Under septic stress, cardiomyocytes package damaged mitochondria or their components into extracellular vesicles for expulsion, a process likely involving MDVs or autophagosome-related vesicles (90). This export may be triggered by severe mitochondrial membrane depolarization and the accumulation of oxidized components, serving as a protective measure against catastrophic intracellular DAMP release. These expelled vesicles are recognized and internalized primarily by TREM2^hi^ macrophages. TREM2 is thought to bind to damage-associated ligands, such as anionic phospholipids, on the vesicle surface (22, 91). This recognition is potentially augmented by other phagocytic receptors detecting signals like phosphatidylserine. The engagement of these receptors triggers the phagocytosis of the vesicle, leading to lysosomal degradation of its dysfunctional cargo. The failure of this clearance pathway—due to loss of TREM2^hi^ macrophages, receptor deficiency, or an overwhelming burden of debris—has dire consequences. Accumulated extracellular mitochondrial material can rupture, releasing potent DAMPs including mtDNA. mtDNA can activate innate immune sensors such as Toll-like receptor 9 (TLR9) and the cytosolic cGAS-STING pathway in various cardiac cells, fueling a feedforward loop of interferon and cytokine production (92). Furthermore, undegraded mitochondrial components internalized by other cells can activate the NLRP3 inflammasome, leading to caspase-1-dependent maturation of IL-1β and IL-18 (47). This creates a vicious cycle of inflammation and oxidative stress. Ultimately, this microenvironment directly impairs cardiomyocyte function by exacerbating oxidative damage to contractile proteins and ion channels, and by disrupting calcium handling, thereby directly linking failed intercellular waste management to the contractile dysfunction characteristic of SRMI (22). While the cardioprotective role of TREM2^hi^ macrophages in sepsis is established (22), the precise identity of TREM2 ligands on mitochondrial vesicles and the full repertoire of cooperating phagocytic receptors remain active areas of investigation, offering promising avenues for therapeutic intervention.

Furthermore, endoplasmic reticulum (ER) stress is closely linked to mitochondrial dysfunction. The interaction between the ER and mitochondria is a vital and complex physiological process critical for normal cellular function and homeostasis. RMDN3, a key tethering protein at ER-mitochondria contact sites, upon phosphorylation with PTPIP51, enhances the tightness of ER-mitochondria membrane contacts, acting as a “molecular channel” for the transfer of lipid radicals from mitochondria to the ER for clearance. In cell models with defective RMDN3/PTPIP51 phosphorylation, the efficiency of lipid radical transfer is reduced, mtROS levels increase, and cellular tolerance to oxidative stress is significantly impaired (83). Another study indicated that dysregulated DUSP1-PDHB2 expression is a key upstream regulatory mechanism for dysfunctional mitochondrial quality control and ER-phagy (93). The communication between mitochondria and other organelles also plays a crucial role in establishing compartmentalized metabolic homeostasis (94). Specifically, interactions with lysosomes regulate iron metabolism and cholesterol transport, those with lipid droplets are vital for lipogenesis and fatty acid oxidation (FAO), and contacts with peroxisomes manage very-long-chain fatty acid oxidation and reactive oxygen species (ROS) detoxification. Research into these localized regulatory mechanisms is fundamental to understanding the precise control of metabolic pathways (94).

### 4.3 Intercellular communication: EVs

The coordination of immunometabolism relies on efficient intercellular communication. As an important component of EVs, Exosomes (30–100 nm) are nanoparticles rich in mRNA, miRNA, DNA, and proteins, released extracellularly upon the fusion of multivesicular bodies (MVBs) – which contain intraluminal vesicles (ILVs) formed by inward budding of the endosomal membrane – with the plasma membrane (95). EVs facilitate communication via a “paracrine” effect and play a significant role in SRMI (95, 96). Studies show that miR-29b-3p in macrophage-derived exosomes can be transferred to cardiomyocytes, where it directly recognizes and binds the 3’UTR of glycogen synthase kinase-3β (GSK-3β), inhibiting its expression and promoting cardiomyocyte autophagy by regulating the phosphorylation level at the GSK-3β Ser9 site (97). miRNA-141 secreted by bone marrow mesenchymal stem cells (BMSC)-derived exosomes (BMSC-Exo) targets PTEN and activates β-catenin, alleviating myocardial injury in septic mice (98). Placental mesenchymal stem cell-derived exosomes loaded with miR-126a-3p inhibit sepsis-induced cardiomyocyte apoptosis and pyroptosis by targeting PIK3R2, activating AKT, and inhibiting the NF-κB pathway (99). BMSC-Exo and their cargo miRNAs can influence macrophage M2 polarization, thereby affecting the progression of sepsis (100–102). However, the precise mechanisms by which key active molecules within BMSC-Exo promote M2 polarization to alleviate SRMI remain unclear and require further investigation.

### 4.4 Vascular endothelium: amplifier and participant in immunometabolism

The intricate interplay between immune dysregulation, endothelial cell dysfunction, and coagulopathy is a key factor in the pathogenesis of sepsis and multiple organ failure (11). In sepsis-related myocardial injury, endothelial cells play an important role in mediating myocardial dysfunction, primarily affecting myocardial relaxation, thrombus formation, and microvascular permeability (103). In response to oxidative stress, endothelial cells synthesize and accumulate ROS, which damage DNA, proteins, and lipids. Blocking iNOS activity has been proposed as a therapeutic strategy to improve microvascular blood flow in septic patients (12). Activation of the NF-κB and NLRP3 pathways in endothelial cells during sepsis further amplifies systemic inflammation and multiple organ dysfunction syndrome (MODS), increasing the expression of TNF-α, IL-6, MCP-1, IFN-γ, iNOS, intercellular cell adhesion molecule-1 (ICAM-1), and vascular cell adhesion molecule-1 (VCAM-1) (12). Endothelial barrier failure in sepsis involves dysregulation of molecular chaperones and cytoskeletal dynamics. Heat shock protein 90 (HSP90), a key molecular chaperone, stabilizes numerous client proteins involved in inflammatory signaling. Pharmacological inhibition of HSP90 has been shown to protect against inflammatory endothelial barrier dysfunction, in part by disrupting RhoA signaling, suggesting a complex role in regulating microvascular integrity during systemic inflammation (104, 105). Endothelial cells share common traits and possess plasticity, but also exhibit organ-specific characteristics; for instance, the distribution of tight and adherens junctions varies significantly across the vascular tree, which may partly explain the phenotypic and outcome differences observed in sepsis originating from different sources (106). Research indicates that Spock2 expression is elevated in a time-dependent manner in cardiac endothelial cells during sepsis-related myocardial injury. Compared to S100A8/A9 and XDH, SPOCK2 may act as a responsive protective factor, as its deficiency exacerbates LPS-induced apoptosis and necrosis (107). Epitranscriptomic regulation, such as N6-methyladenosine (m6A) RNA modification, is an emerging layer of control in cardiovascular pathophysiology. m6A modulates the stability, translation, and processing of mRNAs encoding proteins critical for inflammation, metabolism, and cell survival. Alterations in m6A dynamics have been implicated in heart failure and may influence the response of cardiac and endothelial cells to inflammatory stress, representing a novel frontier in understanding the molecular underpinnings of SRMI (108, 109).

In summary, the immunometabolic crosstalk in SRMI constitutes a multi-layered, highly interconnected network. Signaling molecules generated by metabolic reprogramming directly regulate immune responses; immune cells help maintain tissue homeostasis by clearing dysfunctional organelles; and intercellular communication integrates these components into a coordinated overall response. A deeper understanding of this complex dialogue is crucial for developing innovative therapies targeting immunometabolic imbalance.

## 5 Therapeutic targeting of macrophage metabolism

**Figure 4:**
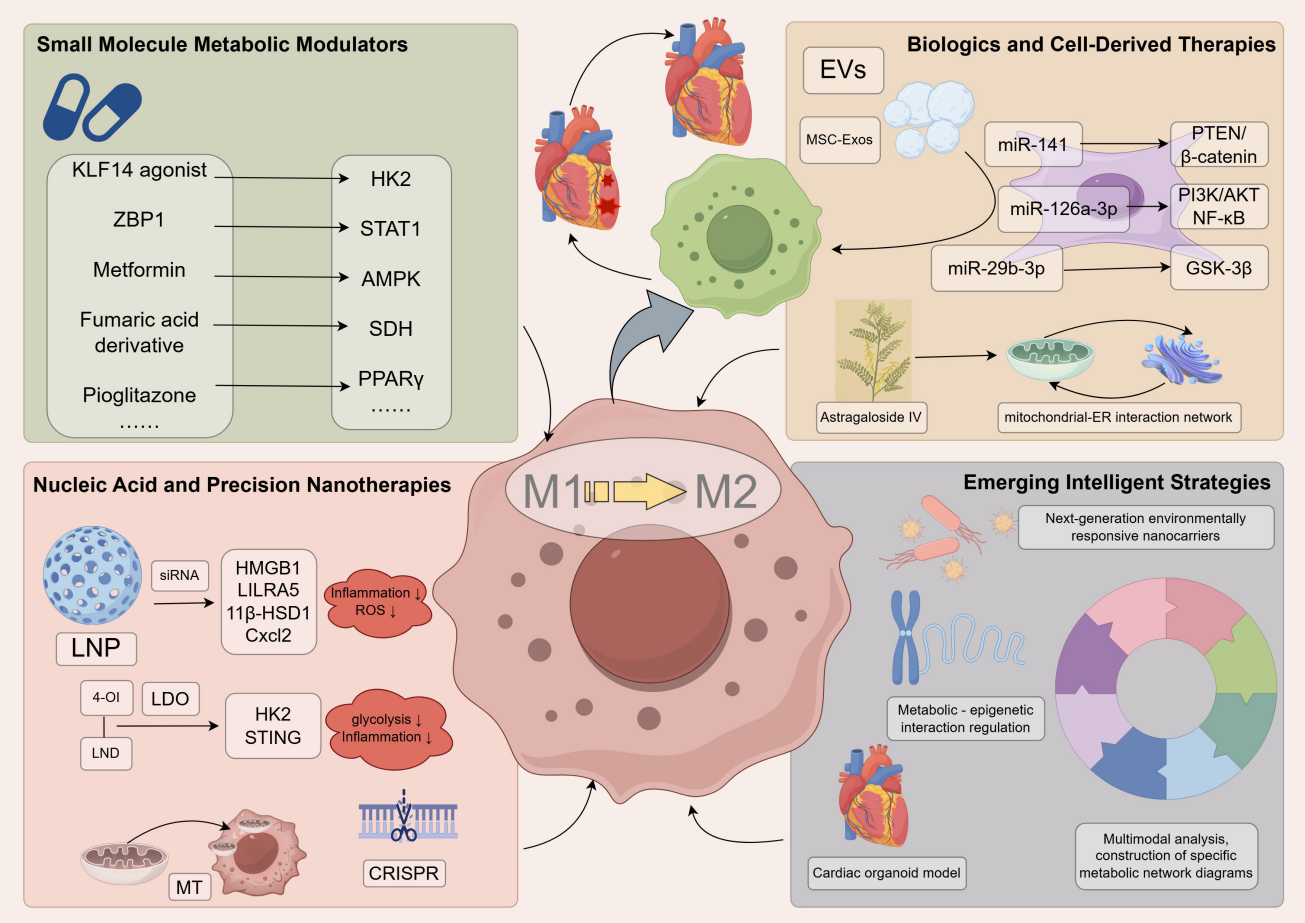
Therapeutic strategies targeting macrophage metabolic reprogramming in sepsis-related myocardial injury. This overview categorizes interventions based on their action within a therapeutic logic model. The cause (therapeutic intervention) is designed to mechanistically modulate specific nodes in macrophage metabolism (e.g., via small molecules, nucleic acids, or biologics). This precise targeting aims to shift the macrophage phenotype from a detrimental M1 state toward a protective M2 state. The intended outcome of these strategies is the attenuation of myocardial injury and the improvement of cardiac function in sepsis. 11β-HSD1, 11β-hydroxysteroid dehydrogenase type 1; 4-OI, 4-octyl-itaconate-lonidamine disulfide; AMPK, AMP-activated Protein Kinase; CRISPR, Clustered Regularly Interspaced Short Palindromic Repeats; Cxcl2, C-X-C Motif Chemokine Ligand 2; EVs, Extracellular Vesicles; GSK-3β, Glycogen Synthase Kinase-3β; HK2, Hexokinase 2; HMGB1, High Mobility Group Box 1; KLF14, Kruppel-like Factor 14; LNP, Lipid Nanoparticle; LILRA5, Leukocyte Immunoglobulin-Like Receptor Subfamily A Member 5; miR, micro RNA; MT, Mitochondrion; NF-κB, Nuclear Factor Kappa-Light-Chain-Enhancer of Activated B Cells; PPARγ, Peroxisome Proliferator-Activated Receptor Gamma; PTEN, Gene of Phosphate and Tension Homology Deleted on Chromosome Ten; ROS, Reactive Oxygen Species; SDH, Succinate Dehydrogenase; siRNA, Small interfering RNA; STAT, Signal Transducer and Activator of Transcription; ZBP1, Z-DNA Binding Protein 1 Gene.

The intricate connection between macrophage metabolism and function during SRMI provides a molecular rationale for exploring novel therapeutic strategies. The current research focus is increasingly directed at the targeted modulation of macrophage metabolism, with the aim of restoring cardiac immune homeostasis. Based on differences in mechanisms of action and therapeutic approaches, we categorize these strategies into four major classes: small molecule metabolic modulators, nucleic acid and precision nanotherapies, biologics and cell-derived therapies, and emerging therapeutic strategies (**Figure 4**).

To provide a synthesized and critical overview of the diverse therapeutic strategies discussed below, we have compiled a comparative summary of key interventions, their molecular targets, preclinical evidence, and translational status (**Table 2**). This table underscores the promising yet preliminary nature of most approaches, which remain predominantly in the experimental domain.

**Table 2:** Summary of emerging therapeutic strategies targeting macrophage metabolism in preclinical models of sepsis-related myocardial injury.

Intervention	Primary molecular target/pathway	Mechanistic effect on macrophage phenotype/metabolism	Model(s) tested	Key cardiac endpoints	Translational status & notes
Small molecule modulators					
KLF14 agonist (64)	HK2 transcription/Glycolysis	Inhibits glycolysis; reduces pro-inflammatory cytokine release	CLP (mouse)	↑ Survival, ↓ atherosclerosis	Preclinical. Novel target; long-term safety unknown.
Metformin (115)	AMPK activation	Suppression of TLR4 activity.	LPS, CLP (rats)	Improved LV function, ↓ inflammatory markers	Repurposed drug. Clinical trials in sepsis show mixed results; cardiac-specific effects unclear.
Pioglitazone (72)	PPARγ agonist	Inhibit AP-1 protein, increase Bcl-2 protein expression, reduce oxidative stress	CLP (rats)	Attenuated cardiac dysfunction, ↓ myocardial hypertrophy, ↓ cardiomyocyte apoptosis.	Approved for diabetes. Risk of fluid retention/heart failure may limit use in sepsis.
4-Octyl-itaconate (4-OI) derivative (67)	Nrf2 activation/inhibit NLRP3	Suppresses glycolysis; reduces pro-inflammatory mediators; promotes resolution	LPS, CLP (mouse)	↓ Myocardial injury markers, ↑ survival	Preclinical. Itaconate derivative with improved pharmacokinetics.
Nucleic acid & nanotherapies					
LNP-siRNA targeting HMGB1 (117)	HMGB1 gene silencing	Reduces release of key DAMP, attenuating inflammation	LPS-induced ALI (mouse)	↓ Cardiac HMGB1, improved systemic hemodynamics	Preclinical. Demonstrates proof-of-concept for targeted siRNA delivery to immune cells.
LNP-siRNA targeting Cxcl2 (33)	Cxcl2 (macrophage chemokine)	Reduces neutrophil chemotaxis; may indirectly alter macrophage activity	Myocardial ischemia (mouse) post-systemic inflammation	↓ Myocardial neutrophil infiltration, ↓ infarct size	Preclinical. Highlights role of macrophage-derived chemokines in secondary injury.
Self-assembled nano-system LDO (4-OI+Lonidamine) (119)	Glycolysis & STING pathway	Synergistically reprograms metabolism; shifts balance from M1 to M2	LPS (macrophages *in vitro*)	Not reported for *in vivo* cardiac models yet	Early preclinical. Innovative dual-action nanotherapy; requires *in vivo* validation in sepsis models.
Biologics & cell-derived therapies					
MSC-derived exosomes (miR-141) (98)	PTEN/β-catenin pathway	Promotes M2 polarization; anti-inflammatory	CLP (mouse)	↓ Myocardial apoptosis, ↓ serum CK-MB and LDH	Preclinical. Cell-free therapy; scalable production and standardization are challenges.
MSC-derived exosomes (miR-126a-3p) (99)	PIK3R2/AKT/NF-κB pathway	Inhibits cardiomyocyte apoptosis/pyroptosis; may modulate microenvironment	LPS, CLP (mouse)	Improved EF, ↓ myocardial apoptosis	Preclinical. Specific cargo-mediated effect; targeting efficiency *in vivo* needs optimization.
Dexmedetomidine (via exosomal miR-29b-3p) (97)	miR-29b-3p/GSK-3β axis	Modulates macrophage-cardiomyocyte crosstalk; promotes cardiomyocyte autophagy	CLP (mouse)	Attenuated cardiac dysfunction, ↓ cTnI	Repurposed drug. Mechanistic link to macrophage metabolism is indirect.

This table synthesizes representative therapeutic interventions currently under preclinical investigation for sepsis-related myocardial injury (SRMI) that target macrophage immunometabolism. It provides a comparative overview of their molecular targets, demonstrated mechanistic effects on macrophage phenotype, the experimental models used for validation, key cardiac functional endpoints measured, and an appraisal of their current translational status. This summary highlights the diversity of approaches and underscores the preclinical stage of this promising field. The numbers listed under the “Intervention” column represent the order of the cited references in the main text.

AMPK, AMP-activated protein kinase; CLP, cecal ligation and puncture; EF, ejection fraction; LNP, lipid nanoparticle; LPS, lipopolysaccharide; LV, left ventricular; MSC, mesenchymal stem cell; NF-κB, nuclear factor kappa-light-chain-enhancer of activated B cells; siRNA, small interfering RNA. KLF14, Kruppel-like Factor 14; HK2, Hexokinase 2; TLR4, Toll-Like Receptor 4; PPARγ, Peroxisome Proliferator-Activated Receptor Gamma; AP-1, Activator Protein-1; Bcl-2, B-cell Lymphoma-2; Nrf2, Nuclear Factor Erythroid 2-Related Factor 2; NLRP3, NLR Family Pyrin Domain Containing 3; Cxcl2, C-X-C Motif Chemokine Ligand 2; STING, Stimulator of Interferon Genes; PTEN, Phosphatase and Tensin Homolog; CK-MB, Creatine Kinase MB Form; LDH, Lactate Dehydrogenase; PIK3R2, Phosphoinositide-3-Kinase Regulatory Subunit 2; AKT, Protein Kinase B; GSK-3β, Glycogen Synthase Kinase-3β; cTnI, Cardiac Troponin I.

↑, increase/upregulation; ↓, decrease/downregulation.

As illustrated in **Table 2**, the field is rapidly exploring interventions that precisely target nodes within macrophage immunometabolism. The current evidence is compelling but rests almost exclusively on preclinical models, primarily murine LPS or CLP paradigms. While these studies consistently show improvements in surrogate cardiac endpoints (e.g., biomarkers, echocardiographic function) and survival, critical translational gaps remain. These include a frequent lack of dose-response relationships, limited assessment of long-term cardiac recovery, and insufficient evaluation in models of pre-existing comorbidities or aging—factors that define the typical septic patient. Furthermore, the organ-specificity and potential off-target effects of metabolic interventions, particularly small molecules and nucleic acid therapies, require thorough investigation. Bridging these gaps will be essential to advance the most promising strategies toward clinical evaluation.

### 5.1 Small molecule metabolic modulators

Small molecule metabolic modulators, which directly regulate metabolic enzyme activity or related signaling pathways, demonstrate significant potential for clinical translation. Suppressing hyperactive glycolysis is a key strategy to attenuate detrimental M1 polarization (64, 110–112). Regarding glycolysis pathway targeting, studies have found that agonists of the transcription factor KLF14 effectively restrict macrophage glycolysis and reduce pro-inflammatory cytokine release by inhibiting HK2 transcription, thereby improving survival in septic mice (64). Furthermore, activating transcription factor 4 (ATF4) can interact with both HK2 and HIF-1α, collectively reinforcing the pro-inflammatory response and influencing immune tolerance (112). Z-DNA binding protein 1 (ZBP1) drives macrophage M1 polarization via a STAT1-dependent mechanism; targeting ZBP1 or its upstream regulator STAT1 can effectively reverse the inflammatory phenotype and improve cardiac function (113). Targeting the glucose transporter GLUT1 is also considered a potential strategy for controlling severe inflammation, such as in sepsis (114). On the other hand, enhancing OXPHOS and FAO, upon which M2 macrophages rely, helps promote repair functions (110, 111). Functioning as a central energy sensor, AMPK is a pivotal regulator of mitochondrial homeostasis, governing processes from mitochondrial generation to clearance via mitophagy. Its activation is universally triggered by perturbations that elevate the AMP/ATP ratio, a common consequence of mitochondrial damage induced by agents such as respiratory chain inhibitors (e.g., metformin), ATP synthase inhibitors, or mtDNA mutations (71). In the context of enhancing oxidative metabolism for sepsis treatment, metformin—by inducing mild energetic stress and activating AMPK—coordinatedly dampens NF-κB activity and pro-inflammatory cytokine networks, an effect consistently demonstrated across multiple sepsis models (115). Similarly, the PPARγ agonist pioglitazone, by enhancing FAO, significantly improves sepsis-associated organ dysfunction, a process involving the activation of mitochondrial biogenesis and the antioxidant defense system (72). Direct supplementation of immunomodulatory metabolites is also an effective approach; for instance, itaconate derivatives can exert anti-inflammatory and cytoprotective effects via activation of the Nrf2 pathway (67). In the context of viral infection, the aspartate-argininosuccinate shunt driven by ASS1 enzyme provides fumarate to macrophages, suggesting its potential as a target for metabolic intervention (116). However, the translational path for these modulators is fraught with challenges, including a potentially narrow therapeutic window that may differ between hyperinflammatory and immunosuppressive sepsis phases, and the risk of off-target effects given the ubiquitous nature of core metabolic pathways.

### 5.2 Nucleic acid and precision nanotherapies

The rapid development of nucleic acid and precision nanotherapies offers unprecedented specificity for metabolic immunotherapy. Gene silencing strategies utilize meticulously designed nanocarriers for targeted delivery. For example, lipid nanoparticles encapsulating HMGB1-specific siRNA effectively block the acute inflammatory response (117). Silencing the specific hub gene LILRA5 significantly attenuates the macrophage-mediated oxidative stress burst in early sepsis (32). Targeted knockdown of 11β-HSD1 activates the AMPK/SIRT1/PGC-1α pathway, alleviating LPS-induced myocardial dysfunction, inflammation, and mitochondrial oxidative stress (118). Using lipid nanoparticle-encapsulated siRNA to target and silence macrophage Cxcl2 markedly reduces myocardial neutrophil infiltration, diminishes inflammation, and reduces infarct size, concurrently demonstrating that aberrant macrophage chemotactic activity is a key target exacerbating post-inflammatory myocardial ischemic injury (33). In recent years, the design concept of intelligent nano-systems has become increasingly sophisticated. The self-assembled nano-system LDO (4-octyl-itaconate-lonidamine disulfide) ingeniously integrates an itaconate derivative with the glycolytic inhibitor lonidamine, rebuilding macrophage polarization balance in complex inflammatory environments by synergistically modulating glycolytic flux and the STING signaling pathway (119). Research indicates that LPS-activated M1 macrophages suffer from mitochondrial dysfunction (e.g., elevated mROS, calcium overload). Although mitochondrial transplantation could conceptually restore function and repolarize macrophages, its practical application faces challenges in targeting and efficiency. One innovative solution involves a “decoupling-assisted partial mitochondrial transplantation” strategy using a pHLIPs-PEG-TPP-Mito (PPT-Mito) complex (120). This construct targets the M1 cell’s acidic surface, and the self-removing pHLIP prevents long-term modification effects. The resulting suppression of glycolytic and PPP pathways favors M2 polarization, offering a novel targeted approach for mitochondrial therapy in sepsis. These advanced delivery systems not only address the targeting issues of traditional therapies but also significantly improve treatment safety through controlled release properties. More encouragingly, emerging CRISPR gene-editing technology is being applied to macrophage metabolic reprogramming, offering the potential for long-lasting immunometabolic balance by precisely regulating the expression of key metabolic enzyme genes.

### 5.3 Biologics and cell-derived therapies

Biologics and cell-derived therapies have opened new dimensions in metabolic immunomodulation. Exosome therapy, as a highly promising cell-free strategy, holds the unique advantage of mimicking natural intercellular communication. Mesenchymal stem cell-derived exosomes (MSC-Exos) act as pleiotropic signal carriers, capable of simultaneously delivering multiple therapeutic miRNAs and regulating complex metabolic networks. Research shows that miR-141 in BMSC-Exos significantly ameliorates the pathological progression of SRMI by finely tuning the PTEN/β-catenin axis (98). Similarly noteworthy, miR-126a-3p, through multi-target regulation of the PI3K/AKT and NF-κB signaling networks, not only inhibits cardiomyocyte death but also promotes the formation of a tissue-reparative microenvironment (99). Dexmedetomidine modulates exosome-mediated macrophage-cardiomyocyte crosstalk via the miR-29b-3p/GSK-3β axis, thereby alleviating sepsis-related myocardial injury (97). In the study of natural active components, Astragaloside IV exhibits unique metabolic regulatory properties; it modulates immune responses while maintaining cellular homeostasis by coordinating the mitochondrial-ER interaction network, providing important insights for developing novel metabolic immunomodulators from traditional medicines (93). A common feature of these biological therapies is their ability to mimic the body’s natural regulatory mechanisms, thereby achieving more precise and safer immunometabolic reprogramming.

### 5.4 Emerging therapeutic strategies

Advances in understanding macrophage metabolic reprogramming are shaping the concept of precision therapeutics in this field. The integration of multi-omics technologies, particularly single-cell transcriptomics and metabolomics, enables the construction of patient-specific metabolic network maps by identifying distinct macrophage subpopulations and their functional states in the SRMI, providing a molecular basis for patient stratification (22, 33). Concurrently, sophisticated intelligent nanocarriers—including pH-sensitive, enzyme-activated, and ROS-responsive systems—have been engineered to achieve spatiotemporally controlled drug release within the pathological myocardial microenvironment, significantly enhancing targeting precision while minimizing systemic toxicity (119, 120). The emergence of human cardiac organoids represents a transformative model system, offering a biomimetic human-derived platform to dissect species-specific macrophage-cardiomyocyte crosstalk and evaluate novel therapeutics within a pathophysiologically complex yet controlled microenvironment, and artificial intelligence plays a supporting role in it (121). Furthermore, the mechanistic understanding of metabolite-mediated epigenetic reprogramming continues to expand, with evidence showing that lactate-induced histone lactylation and itaconate-driven Nrf2 activation can establish and sustain an anti-inflammatory macrophage phenotype, opening avenues for durable immunomodulation (78, 82, 84). The convergence of these technologies—deep phenotyping, intelligent delivery, human-relevant modeling, and epigenetic editing—heralds a new era of multi-dimensional, systems-level interventions for SRMI, moving beyond the constraints of single-target inhibition.

## 6 Conclusions and future perspectives

### 6.1 Study limitations and challenges

Although current research has significantly deepened our understanding of the role of macrophage metabolic reprogramming in SRMI, several important limitations persist. Firstly, the majority of mechanistic evidence is derived from genetically modified mouse models and cell co-culture systems. While these models provide invaluable mechanistic insights, their translational relevance to the complex pathophysiology of human sepsis requires careful evaluation. Human sepsis is highly heterogeneous, and existing animal models struggle to fully recapitulate this diversity. Secondly, while current spatial omics technologies can decipher the metabolic signatures of cell subpopulations, techniques for simultaneously analyzing metabolite flux and protein activity at the single-cell level remain underdeveloped, limiting our comprehensive understanding of dynamic metabolic processes. Furthermore, most studies focus on short-term metabolic alterations; the sustained effects of metabolic reprogramming during the chronic phase of SRMI and its long-term impact on cardiac repair remain unclear. Critically, the level of evidence supporting the individual mechanisms discussed varies. The pathophysiological roles of key metabolites (e.g., succinate, lactate) and signaling pathways (e.g., TLR4/NF-κB) are supported by extensive preclinical data and corroborative human biomarker studies (66, 78, 81). In contrast, more recently elucidated mechanisms such as macrophage-mediated ferroptosis or the therapeutic potential of sophisticated nano-therapies, while compelling in rodent models, remain primarily hypothesis-generating in the specific context of human SRMI and await validation in clinical cohorts (49, 119). Consequently, while existing therapeutic strategies show promise in pre-clinical models, significant challenges such as organ specificity, potential off-target effects, and inter-individual metabolic variability must be addressed prior to clinical translation. Finally, while this review focuses on the immunometabolic axis centered on macrophages, it is a deliberate synthesis of a dominant but not exclusive pathway. The relative contribution of this axis compared to other immune cell-driven processes or direct microbial effects in human SRMI remains an open question, marking a significant boundary for the inferences drawn herein.

### 6.2 Future research directions

Addressing the current limitations necessitates a focused effort on several key fronts in future research. On the technological innovation front, developing novel analytical platforms capable of integrating multi-omics data, combined with AI-driven methods to construct dynamic metabolic network models, will help unveil the temporal patterns and compensatory mechanisms of metabolic reprogramming. Regarding model systems, establishing organoid-immune cell co-culture systems incorporating human genetic backgrounds, coupled with organ-on-a-chip technology to mimic the cardiac microenvironment, will provide more reliable platforms for studying human-specific immunometabolic interactions. In the clinical translation pathway, prospective cohort studies in septic patients are needed to systematically analyze the association between immune cell metabolic profiles and the evolution of cardiac function, thereby informing personalized treatment strategies. Concurrently, the optimization of intelligent delivery systems should prioritize resolving targeting efficiency and biosafety issues to facilitate the translation of metabolic intervention strategies into clinical applications.

### 6.3 Conclusions

This review has systematically elaborated on the central role of macrophage metabolic reprogramming in SRMI, establishing metabolic regulation as a critical bridge connecting the immune response to cardiac function. Existing evidence indicates that the transition between macrophage polarization states is fundamentally determined by their metabolic status—the glycolytic flux characteristic of the M1 phenotype and the OXPHOS/FAO hallmark of the M2 phenotype not only provide the energetic basis but also directly participate in signal transduction and epigenetic regulation via metabolites (e.g., succinate, itaconate), forming a sophisticated immunometabolic network. Targeting these metabolic nodes (e.g., using itaconate derivatives or engineered nanozymes) has demonstrated therapeutic potential for rebuilding immune homeostasis in pre-clinical studies, signifying a shift in therapeutic strategy from broad-spectrum immunosuppression towards precise metabolic intervention.

As these scientific questions are progressively answered, accumulating preclinical evidence provides a rationale to explore therapeutic strategies targeting macrophage metabolism as potential future avenues for the clinical management of SRMI. Future research should be dedicated to bridging the chasm between mechanistic understanding and clinical application. Through multidisciplinary collaboration, we can propel the in-depth development of metabolic immunology in the field of critical care medicine, ultimately advancing towards a shift in the medical paradigm from “disease treatment” to “health restoration”.

## Glossary

11β-HSD1: 11β-Hydroxysteroid Dehydrogenase Type 1
ACSL4: Acyl-CoA Synthetase Long-Chain Family Member 4
ADP: Adenosine Diphosphate
AMPK: AMP-Activated Protein Kinase
Arg1: Arginase 1
ATP: Adenosine Triphosphate
BMSC: Bone Marrow Mesenchymal Stem Cell
CCL: C-C Motif Chemokine Ligand
CCR2: C-C Motif Chemokine Receptor 2
CLP: Cecal Ligation and Puncture
CPT1: Carnitine Palmitoyltransferase1
CRISPR: Clustered Regularly Interspaced Short Palindromic Repeats
Cxcl2: C-X-C Motif Chemokine Ligand 2
DAMP: Damage-Associated Molecular Pattern
ER: Endoplasmic Reticulum
EVs: Extracellular Vesicles
Exo: Exosomes
FADH2: Flavine Adenine Dinucleotide, Reduced
FAO: Fatty Acid Oxidation
FFA: Free Fat Acid
GABA: Gama-Aminobutyric Acid
GLUT1: Glucose Transporter 1
GPX4: Glutathione Peroxidase 4
GSK-3β: Glycogen Synthase Kinase-3β
HIF-1α: Hypoxia-Inducible Factor 1-alpha
HK2: Hexokinase 2
HLA-DR: Human Leukocyte Antigen DR
HMGB1: High Mobility Group Box 1
HSP90AA1: Heat Shock Protein 90 Alpha Family Class A Member 1
IFN-γ: Interferon-gamma
IL: Interleukin
iNOS: Inducible Nitric Oxide Synthase
JAK: Janus Kinase
KLF14: Kruppel-like Factor 14
Lac: Lactate
LILRA5: Leukocyte Immunoglobulin-Like Receptor Subfamily A Member 5
LPS: Lipopolysaccharide
MAPK: Mitogen-Activated Protein Kinase
MHC-II: Major Histocompatibility Complex Class II
miRNA: MicroRNA
MODS: Multiple Organ Dysfunction Syndrome
MT: Mitochondrion
mtDNA: Mitochondrial DNA
mTOR: Mammalian Target of Rapamycin
mtROS: Mitochondrial Reactive Oxygen Species
NADH: Nicotinamide Adenine Dinucleotide
NADPH: Nicotinamide Adenine Dinucleotide Phosphate Oxidase
NF-κB: Nuclear Factor Kappa-Light-Chain-Enhancer of Activated B Cells
NLRP3: NLR Family Pyrin Domain Containing 3
NO: Nitric Oxide
Nrf2: Nuclear Factor Erythroid 2-Related Factor 2
OXPHOS: Oxidative Phosphorylation
PAMP: Pathogen-Associated Molecular Pattern
PGC-1β: Peroxisome Proliferator-Activated Receptor Gamma Coactivator-1β
PHDs: Prolyl Hydroxylases
PI3K: Phosphatidyqinositol‐3 Kinase
PKM2: M2 Isoform of Pyruvate Kinase
PPARγ: Peroxisome Proliferator-Activated Receptor Gamma
PPP: Pentose Phosphate Pathway
PRR: Pattern Recognition Receptor
RET: Reverse Electron Transport
RNS: Reactive Nitrogen Species
ROS: Reactive Oxygen Species
SRMI: Sepsis-Related Myocardial Injury
STAT: Signal Transducer and Activator of Transcription
TCA: Tricarboxylic Acid
TGF-β: Transforming Growth Factor-Beta
TLR4: Toll-Like Receptor 4
TNF-α: Tumor Necrosis Factor-Alpha
TREM2: Triggering Receptor Expressed on Myeloid Cells 2
ZBP1: Z-DNA Binding Protein 1
α-KG: α-Ketoglutaric Acid
